# Reduced Chlorhexidine and Daptomycin Susceptibility in Vancomycin-Resistant Enterococcus faecium after Serial Chlorhexidine Exposure

**DOI:** 10.1128/AAC.01235-17

**Published:** 2017-12-21

**Authors:** Pooja Bhardwaj, Amrita Hans, Kinnari Ruikar, Ziqiang Guan, Kelli L. Palmer

**Affiliations:** a Department of Biological Sciences, University of Texas at Dallas, Richardson, Texas, USA; b Department of Biochemistry, Duke University Medical Center, Durham, North Carolina, USA

**Keywords:** Enterococcus, chlorhexidine, daptomycin

## Abstract

Vancomycin-resistant Enterococcus faecium strains (VREfm) are critical public health concerns because they are among the leading causes of hospital-acquired bloodstream infections. Chlorhexidine (CHX) is a bisbiguanide cationic antiseptic that is routinely used for patient bathing and other infection control practices. VREfm are likely frequently exposed to CHX; however, the long-term effects of CHX exposure have not been studied in enterococci. In this study, we serially exposed VREfm to increasing concentrations of CHX for a period of 21 days in two independent experimental evolution trials. Reduced CHX susceptibility emerged (4-fold shift in CHX MIC). Subpopulations with reduced daptomycin (DAP) susceptibility were detected, which were further analyzed by genome sequencing and lipidomic analysis. Across the trials, we identified adaptive changes in genes with predicted or experimentally confirmed roles in chlorhexidine susceptibility (*efrE*), global nutritional stress response (*relA*), nucleotide metabolism (*cmk*), phosphate acquisition (*phoU*), and glycolipid biosynthesis (*bgsB*), among others. Moreover, significant alterations in membrane phospholipids were identified for some populations with reduced DAP susceptibility. Our results are clinically significant because they identify a link between serial subinhibitory CHX exposure and reduced DAP susceptibility. In addition, the CHX-induced genetic and lipidomic changes described in this study offer new insights into the mechanisms underlying the emergence of antibiotic resistance in VREfm.

## INTRODUCTION

Enterococcus faecium is a Gram-positive bacterium that normally colonizes the human gastrointestinal tract and is an opportunistic pathogen associated with bacteremia, urinary tract infections, endocarditis, and wound infections (1–3). Vancomycin-resistant E. faecium strains (VREfm) are of particular concern for infection treatment. VREfm are among the primary etiological agents of central line-associated bloodstream infections (CLABSIs), a type of health care-associated infection (HAI) that arises from central venous catheter use and is associated with high mortality in the United States (4, 5). E. faecium contamination on indwelling venous catheters, surgical instruments, and hospital surfaces is challenging to eradicate (2, 6–8). In hospital and clinical settings, improper infection control practices, contaminated surfaces, and indiscriminate use of antibiotics contribute to the persistence of E. faecium (6, 9).

Chlorhexidine (CHX) is a cationic antiseptic and membrane-active antimicrobial (10–12). The primary mechanism of action of CHX is to disrupt the bacterial cell membrane and cause leakage of cytoplasmic contents and precipitation of cytoplasm (13–15). CHX is recommended by the Society for Healthcare Epidemiology of America to reduce CLABSI occurrence in acute care hospitals (16). Specifically, CHX bathing and CHX-impregnated cardiovascular catheters are used for CLABSI control (16–18). Clinical reports have raised concerns about the long-term effects of CHX bathing on hospital-associated pathogens (19–22). The CHX concentrations on patient skin can fall below the MIC for VREfm between bathings (22). Frequent exposure to subinhibitory CHX could select for VREfm mutants with reduced susceptibility to CHX and other antimicrobials that also interact with the bacterial cell surface. It was recently reported that colistin resistance emerged in the Gram-negative pathogen Klebsiella pneumoniae after exposure to CHX (23).

In a previous study, we used RNA sequencing to study the global transcriptomic responses of a VanA-type VREfm strain to CHX (24). We found that CHX exposure elicited expression of genes associated with antibiotic resistance and extracytoplasmic stress, including genes associated with vancomycin resistance (*vanHAX*) and reduced daptomycin (DAP) susceptibility (*liaXYZ*) (24). In the present study, we test the hypothesis that serial exposure to sub-MIC CHX selects for VREfm mutants with reduced susceptibilities to CHX and other membrane and cell wall-targeting antimicrobials, with particular focus on DAP.

## RESULTS

### *In vitro* evolution of reduced CHX susceptibility in VREfm.

Previous RNA sequencing analysis identified up to 118-fold upregulation of *liaXYZ* in CHX-treated E. faecium 1,231,410 (E. faecium 410) (24), a VanA-type vancomycin- and ampicillin-resistant blood isolate and member of the hospital-adapted clade A1 (25, 26). This was of interest because *liaXYZ* is protective against DAP (27–32). We confirmed the CHX-stimulated upregulation of *liaX* in E. faecium 410 and a commensal clade B strain, E. faecium 1,141,733 (26), using reverse transcription-quantitative PCR (RT-qPCR; see Fig. S1 in the supplemental material).

**FIG 1:**
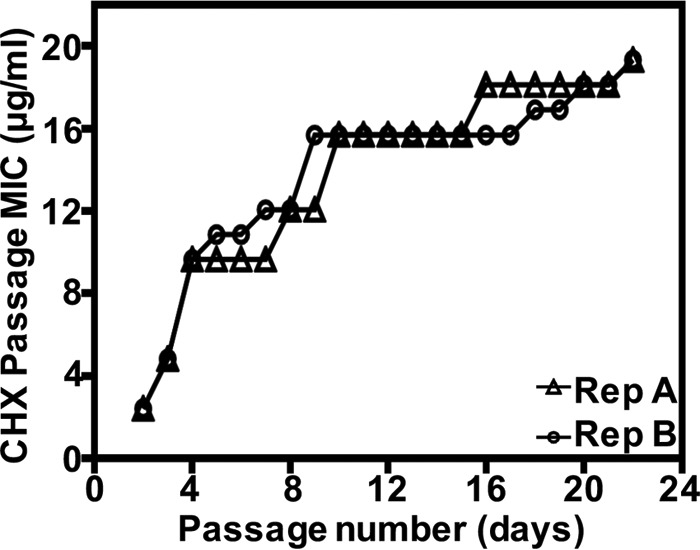
E. faecium adapts to CHX. *In vitro* evolution of reduced CHX susceptibility in E. faecium 1,231,410 (E. faecium 410) by serial passaging in increasing concentrations of CHX for a period of 21 days. CHX passage MIC (*y* axis) for each day of passage (*x* axis) is shown for two independent experiments (A and B).

We hypothesized that repeat exposure to subinhibitory CHX could select for mutants with reduced susceptibility to CHX and concomitant reduced susceptibility to other antimicrobials. To test this, we performed *in vitro* serial passaging of E. faecium 410 with CHX for a period of 21 days, starting with a sub-MIC of 2.9 μg/ml (Fig. 1). Similar patterns of MIC shifts were observed for two independent trials over the course of 21 days. The CHX MICs of the evolved populations (referred to as populations A and B) (Table 1) recovered after one drug-free passage were confirmed to be increased (19.6 μg/ml) compared to the parental strain (Table 2). The CHX MIC was not altered in E. faecium 410 passaged for 21 days in medium without CHX. We conclude that reduced CHX susceptibility emerges in VREfm after serial *in vitro* CHX exposure.

**TABLE 1:** Bacterial strains and plasmids used in this study

Strain or plasmid	Description^*a*^	Source or reference
Bacterial strains		
E. faecium		
1,231,410	Clade A skin and soft tissue infection isolate; VanA-type VRE; DAP^s^	25, 26
1,141,733	Clade B clinical isolate; VAN^s^	25, 26
Population A	*In vitro*-evolved population with reduced CHX susceptibility from expt A, stocked from day 22	This study
Population B	*In vitro*-evolved population with reduced CHX susceptibility from expt B, stocked from day 22	This study
410-P1	E. faecium 410 *in vitro*-evolved population in BHI from day 21, trial 1	This study
410-P2	E. faecium 410 *in vitro*-evolved population in BHI from day 21, trial 2	This study
DAP-A1	Mutants with reduced DAP susceptibility from population A	This study
DAP-A2	Mutants with reduced DAP susceptibility from population A	This study
DAP-B1	Mutants with reduced DAP susceptibility from population B	This study
DAP-B2	Mutants with reduced DAP susceptibility from population B	This study
PB301	E. faecium 410 EFTG_02287-88 deletion mutant	This study
PB302	E. faecium DAP-A2 EFTG_02287-88 deletion mutant	This study
AH101	PB301 transformed with empty pLZ12 plasmid	This study
AH102	PB301 transformed with pAH201	This study
PB303	PB301 transformed with pPB202	This study
E. coli EC1000	E. coli cloning host; provides *repA* in *trans*; F^−^ *araD139*(*ara ABC-leu*)*7679 galU galK* *lacX74 rspL thi*; *repA* of pWV01 in *glgB*; KAN^r^	74
Plasmids		
pLZ12	E. coli and Streptococcus shuttle vector	69
pHA101	Markerless counterselectable exchange plasmid	24
pAH201	pLZ12 containing a 4.186-kb BamHI/BamHI-digested fragment with EFTG_02287-88 and predicted promoter	This study
pPB202	pLZ12 containing a 4.186-kb BamHI/BamHI-digested fragment with EFTG_02287-88 and predicted promoter from strain DAP-A2	This study
pPB301	pHA101 containing a 1.998-kb EcoRI/BamHI-digested fragment with upstream and downstream regions flanking EFTG_02287-88	This study

a VAN^s^, vancomycin susceptible; DAP^s^, daptomycin susceptible; Km, kanamycin.

**TABLE 2:** MIC values for cell wall-targeting antibiotics

Strain or population	MIC (μg/ml)^*a*^			
	CHX	DAP range, median (*P*)	AMP	VAN
E. faecium 410	4.9	2, 2 (NA)	195	250
Population A	19.6	2–6, 3 (0.0085)	390	500
Population B	19.6	3–4, 3.5 (0.0006)	195	500
DAP-A1	19.6	3–6, 4 (0.0043)	195	500
DAP-A2	19.6	3–6, 4 (0.0015)	390	1,000
DAP-B1	19.6	3–4, 3.5 (0.0006)	195	1,000
DAP-B2	19.6	3–4, 3.5 (0.0006)	195	1,000
PB301 (E. faecium 410 Δ*efrEF*)	1.2	2–3, 2 (0.211)	ND	ND
PB302 (DAP-A2 Δ*efrEF)*	2.4	2–4, 3 (0.00188)	ND	ND

a DAP MICs were determined independently either seven times (E. faecium 1,231,410 and population A) or six times (all other determinations). A one-tailed unpaired Student *t* test was used to assess the significance (*P*) of the difference in MIC versus E. faecium 1,231,410. E. faecium 410, E. faecium 1,231,410; AMP, ampicillin; VAN, vancomycin; NA, not applicable; ND, not determined.

Populations A and B had variable but significantly higher DAP MICs relative to the wild-type strain (Table 2), with the DAP MIC in some experimental trials meeting the ≥4 μg/ml breakpoint for DAP resistance (33). The vancomycin MIC was 2-fold higher for both populations relative to the wild type, and ampicillin MIC was 2-fold higher for population A.

### Reduced DAP susceptibility emerges during serial CHX exposure.

**FIG 2:**
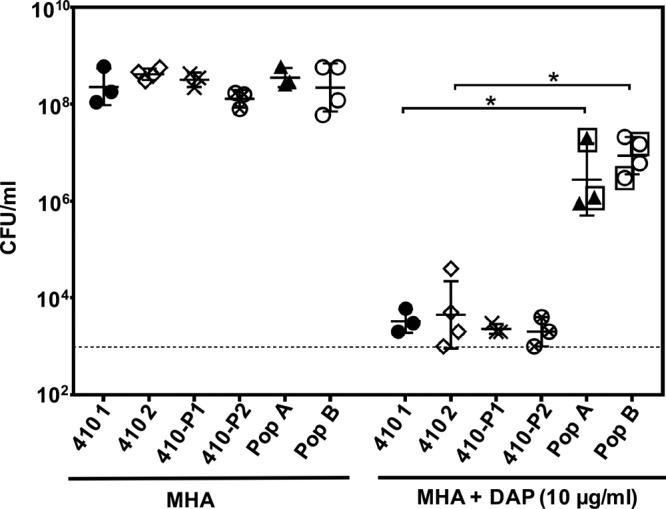
Reduced DAP susceptibility in CHX-passaged E. faecium populations A and B. The geometric mean and the geometric standard deviation of the CFU/ml count for *n* = 3 or 4 independent trials are shown. Strains and populations are described in the text. The dashed line represents limit of detection (10^3^ CFU/ml). The boxed populations were analyzed by whole-genome sequencing. *, *P* < 0.05 (one-tailed Student *t* test).

We sought to further quantify and investigate the basis for the elevated DAP MIC in the CHX-passaged populations. The CHX-passaged populations (A and B) were cultured on agar with or without 10 μg/ml DAP to quantify the CFU (Fig. 2). E. faecium 410 wild-type stock cultures (410-1 and 410-2) and E. faecium 410 passaged for 21 days in the absence of CHX (410-P1 and 410-P2) were used as controls. Subpopulations with reduced DAP susceptibility were detected in the CHX-passaged populations (Fig. 2).

Next, isolated colonies arising on DAP plates from each of two independent DAP plating trials were pooled and stocked for further analysis. These mutants are referred to here as DAP-A1 and DAP-A2 (from population A; 14 and 11 colonies were pooled, respectively) and DAP-B1 and DAP-B2 (from population B; 18 and 5 colonies were pooled, respectively). The DAP MICs of these mutants after two DAP-free passages were found to be variable but were significantly higher than the wild-type E. faecium 410 (Table 2). The DAP mutants, with the exception of DAP-B2, have significantly longer generation times than E. faecium 410 wild-type in BHI broth (see Table S2 in the supplemental material). No significant differences in spontaneous rifampin resistance frequencies were observed between the DAP mutants and wild-type E. faecium 410 (see Table S2 in the supplemental material), indicating that these strains are not hypermutators. RT-qPCR analysis identified a small but statistically significant increase in *liaX* expression in the DAP-A1 and DAP-A2 mutants but not the DAP-B1 and DAP-B2 mutants relative to the wild type (see Fig. S2 in the supplemental material). Recently, a two-component system that contributes to CHX tolerance, referred to as ChtRS, was identified in E. faecium (34). The ChtRS regulon is currently undefined. We identified a small (1.5-fold) but statistically significant increase in *chtS* transcript levels in the DAP-A2 mutant compared to wild-type E. faecium 410 (see Fig. S2 in the supplemental material).

### Mutations in DAP strains.

Genome sequencing identified mutations occurring in the DAP strains relative to wild-type E. faecium 410 (Table 3). All four share a mutation in *efrE* (EFTG_02287), which encodes one subunit of the heterodimeric ABC transporter EfrEF (35). The mutation results in an Ala290Val substitution within the sixth transmembrane helix of EfrE, as predicted by TMHMM 2.0 (36). *efrE* is upregulated 22-fold by E. faecium 410 in response to CHX (24), and deletion of *efrE* from E. faecalis OG1RF confers increased susceptibility to CHX (37, 38). No mutations other than in *efrE* were common to all strains.

**TABLE 3:** Mutations identified by whole-genome sequencing in the DAP-resistant mutants

Mutant	Gene description	Nucleotide variation (frequency [%])^*a*^	Amino acid change^*b*^	E. faecalis V583 ortholog
DAP-A1	Eftg_02287 ABC transporter	C67008T (98.47)	Ala290Val	EF2226
	Eftg_01724 RelA/SpoT (*relA*)	CTAGGATTTAC30479Del (98.19)	Leu505fs	EF1974
	Eftg_01135 alpha/beta hydrolase	G137748A (55.64)	Trp150*	EF1505
	Eftg_00577 cytidylate kinase (*cmk*) promoter	A618699IS*1251*	IS*1251* insertion	EF1547
DAP-A2	Eftg_02287 ABC transporter	C67008T (99.77)	Ala290Val	EF2226
	Eftg_01724 RelA/SpoT (*relA*)	CA29139AG (95.74)	Ala58Glu	EF1974
	Eftg_01135 alpha/beta hydrolase	137847G insertion (88.17)	Ile184fs	EF1505
	Eftg_00577 cytidylate kinase (*cmk*) promoter	A618699IS*1251*	IS*1251* insertion	EF1547
DAP-B1	Eftg_02287 ABC transporter	C67008T (98.962)	Ala290Val	EF2226
	Eftg_01175 PhoU regulator	G173845A (99.61)	Glu61Lys	EF1754
	Eftg_02534 transposase	C71844A (99.47)	Lys206Asn	
DAP-B2	Eftg_02287 ABC transporter	C67008T (99.36)	Ala290Val	EF2226
	Eftg_02162 glycosyltransferase (*bgsB)*	C50223T (67.09)	Val57Met	EF2890

a The mutation frequency in the read assembly was determined by variant detection in CLC Genomics Workbench where applicable. The precise location of insertion for variation A618699IS*1251* was mapped by Sanger sequencing.

b Asterisk indicates stop codon.

DAP-A1 and DAP-A2 share IS*1251* insertions in the promoter region of *cmk*, which encodes cytidylate kinase (Table 3). Population heterogeneity in regard to IS*1251* insertion at the *cmk* promoter was observed in the DAP-A1 and DAP-A2 mutants by gel electrophoresis analysis of *cmk* promoter amplicons (see Fig. S3 in the supplemental material). RT-qPCR confirmed significant downregulation of *cmk* transcript levels in the DAP-A mutants in three independent trials (see Fig. S3 in the supplemental material).

DAP-A1 and DAP-A2 each have different mutations in *relA* and in a gene encoding a predicted alpha/beta hydrolase (Table 3). In DAP-A1, the deletion of 11 bases (CTAGGATTTAC) from *relA* results in synthesis of a truncated, 505-amino-acid protein that lacks the C-terminal ACT regulatory domain. The suggested function of the ACT domain is regulation of the catalytic activities of the amino-terminal domain to ensure that synthesis and degradation of ppGpp are not costimulated (39, 40). In DAP-A2, the A58E substitution is predicted by EMBOSS (41) to convert a beta-strand fold into an alpha helix in the HD4 metal-dependent phosphohydrolase domain of RelA. DAP-A1 and DAP-A2 also possess nonsense and frameshift mutations, respectively, in a gene encoding a predicted alpha/beta hydrolase. To our knowledge, this gene has not previously been linked with antimicrobial susceptibility.

Different mutations were identified in DAP-B1 and DAP-B2. In DAP-B1, an E61K substitution occurs in PhoU, a regulator of phosphate transport. We confirmed that inorganic phosphate concentrations were significantly lower in the DAP-B1 mutant relative to the wild type for two of four time points assayed (see Fig. S4 in the supplemental material).

In DAP-B2, a V57M substitution occurs in BgsB (42). BgsB, a glycosyltransferase (GT), catalyzes the transfer of glucose from UDP-glucose to 1,2-diacylglycerol (DAG) forming 3-d-glucosyl-1,2-diacylglycerol (MGlcDAG), which is converted to diglucosyl-DAG (DGlcDAG) by BgsA (43).

### Distinct alternations of membrane phospholipid compositions in DAP mutants.

We next compared lipid profiles between the DAP mutants and wild-type E. faecium 410 in three independent trials. Ion intensities of phospholipid and glycolipids detected were normalized by wet pellet weight for each lipid extraction, and the normalized levels were compared for wild-type and mutant strains (see Data Set S1 in the supplemental material). Significant changes, as assessed by the one-tailed Student *t* test, are presented in Table 4. Representative negative-ion chromatogram data are shown in Fig. S5 in the supplemental material.

**TABLE 4:** Average fold ratios of significant phospholipid changes in DAP-A1 and DAP-A2 strains

Lipid	Avg fold ratio for lipids detected in DAP strains^*a*^	
	DAP-A1	DAP-A2
Phosphatidylglycerol	−1.41	−1.17
Cardiolipin	−11.28	−6.97
MHDAG (monohexosyldiacylglycerol)	−1.89	
DHDAG (dihexosyldiacylglycerol)	1.89	
GPDD (glycerolphosphate diglucosyl diacylglycerol)		2.07

a The average fold ratios of differences between normalized ion intensities of the DAP mutants were compared to wild-type E. faecium 410 in three independent trials. The ion intensities were normalized by wet pellet weight. A one-tailed Student *t* test was applied to calculate the *P* value.

Cardiolipin (bisphosphatidylglycerol; CL), an anionic phospholipid, was significantly reduced the in DAP-A1 and DAP-A2 mutants compared to E. faecium 410 (Table 4). The expression levels of two predicted cardiolipin synthase (*cls*) genes (*cls*-1 [EFTG_00614] and *cls*-2 [EFTG_01168]), which mediate the reversible transphosphatidylation of PG molecules to synthesize cardiolipin, were not significantly altered compared to E. faecium 410 (see Fig. S2 in the supplemental material). We infer that E. faecium possesses posttranscriptional mechanisms to regulate cardiolipin synthesis and turnover. In the DAP-A1 mutant, the levels of the glycolipids MHDAG and DHDAG were also significantly altered compared to the E. faecium 410 parental strain (Table 4). In the DAP-A2 mutant, the levels of GPDD (glycerolphosphate diglucosyl-diacylglycerol), a lipoteichoic acid precursor, were 2-fold higher (Table 4).

In contrast to DAP-A mutants, lipid profiles of the DAP-B mutants were not significantly altered from the parental strains (see Data Set S1 in the supplemental material), indicating that DAP-A and DAP-B mutants do not share identical molecular mechanisms for reduced DAP susceptibility. We note that CL levels were reduced in every trial for DAP-B2, although this did not achieve statistical significance (*P* = 0.0503; our cutoff for significance is *P* < 0.05; see Data Set S1 in the supplemental material).

### *efrE* impacts CHX but not DAP susceptibility.

**FIG 3:**
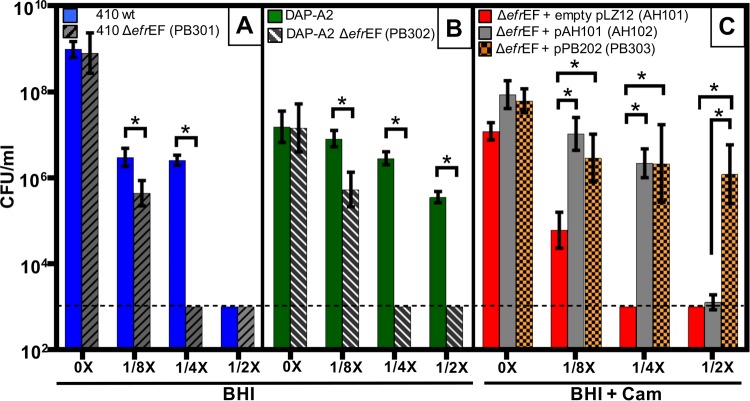
Agar CHX susceptibility assay. The geometric means and the geometric standard deviations of the CFU/ml are shown for wild-type E. faecium 410 and the Δ*efrEF* deletion mutant (A), the DAP-A2 mutant and the DAP-A2 Δ*efrEF* deletion mutant (PB302) (B), and the Δ*efrEF* deletion mutant transformed with empty pLZ12 vector or the complementation vectors pAH201 or pPB202 (C). The CFU/ml value was determined using BHI or BHI-chloramphenicol agars with or without CHX supplementation at 1/8×, 1/4×, or 1/2× MIC CHX. Data from three independent trials are shown for each condition. The dashed line represents the limit of detection (10^3^ CFU/ml). *, *P* < 0.05 value (one-tailed Student *t* test). Cam, chloramphenicol.

Because *efrE* mutations were shared by all DAP strains, we deleted the EfrEF transport system from E. faecium 410 and the DAP-A2 mutant to assess its role in CHX and DAP susceptibility. The deletion of *efrEF* from both E. faecium 410 and the DAP-A2 mutant resulted in increased CHX susceptibility, as determined by broth microdilution assays (Table 2). We also utilized a spot-plating assay to assess CHX susceptibility in the presence of different concentrations of CHX (Fig. 3). As expected, the *efrEF* deletion mutants were significantly more susceptible to CHX than their parent strains.

To complement the *efrEF* deletion in *trans*, we first cloned *efrEF* from wild-type E. faecium 410, along with its predicted native promoter, into plasmid pLZ12 and then transformed it into the *efrEF* deletion mutant, generating strain AH102. As a control, empty pLZ12 vector was also transformed into the *efrEF* deletion mutant, generating strain AH101. We utilized the spot-plating assay to quantify CHX susceptibility differences between strains AH101 and AH102 in the presence of chloramphenicol selection for pLZ12 (Fig. 3). Complementation with the wild-type *efrEF* genes in *trans* significantly reduced the CHX susceptibility of the *efrEF* mutant (Fig. 3).

Next, to assess the contribution of the EfrE Ala290Val substitution in CHX susceptibility, we cloned *efrEF* with its predicted native promoter from the DAP-A2 mutant into the plasmid pLZ12 to generate a second complementation construct, pPB202. This plasmid was transformed into the *efrEF* deletion mutant to generate strain PB303. This construct significantly reduced the CHX susceptibility of the *efrEF* deletion mutant (Fig. 3) and provided greater protection from CHX than the wild-type *efrEF* genes (see data for 1/2× MIC CHX cultures in Fig. 3).

We assessed DAP MICs for the *efrEF* deletion mutants. The DAP MIC of the E. faecium 410 Δ*efrEF* mutant was not significantly different from the wild-type parent (Table 2). For the DAP-A2 Δ*efrEF* mutant, the range and median of the DAP MICs were lower than for DAP-A2, but this change was not significant (*n* = 6 trials; *P* = 0.07 in a one-tailed unpaired Student *t* test).

We performed comparative lipidomic analysis of E. faecium 410 and the *efrEF* deletion mutant in two independent trials. Data for anionic membrane phospholipid and glycolipid levels are shown in Data Set S2 in the supplemental material; note that we did not perform a statistical analysis because only two trials were performed. The most striking differences observed were for two species whose positive ion [M+H]^+^ signals are detected at *m/z* 288 and *m/z* 316 by electrospray ionization/mass spectrometry (ESI/MS). These two species are present in wild-type E. faecium 410 but undetected in the *efrEF* deletion mutant (see Fig. S6 in the supplemental material). High-resolution mass measurement and tandem MS analysis identified these species as ethoxylated fatty amines previously identified as components of the anti-static additive Atmer-163 (44), which is commonly used in consumer products such as plastics. The *m/z* 288 and 316 ions correspond to the [M+H]^+^ ions of Atmer-163 containing C_13_ and C_15_ fatty alkyl chains, respectively. The absence of Atmer-163 in the *efrEF* mutant suggests that this ABC transporter is involved in transporting the Atmer-163 species, with lipid-like structures, from media into the cells.

## DISCUSSION

The goal of this study was to test the hypothesis that serial exposure to sub-MIC CHX selects for VREfm mutants with reduced susceptibilities to CHX, with concomitant effects on susceptibility to other membrane- and cell wall-targeting antimicrobials. Our serial passage experiments demonstrate that reduced CHX susceptibility emerges in VREfm after repeat subinhibitory exposure. Moreover, our DAP plating experiments demonstrate that reduced DAP susceptibility concomitantly emerges in a subpopulation of CHX-passaged cells. Using genomics and lipidomics, we identified genetic and physiological changes occurring in these subpopulations with reduced DAP susceptibility. That the strains arising on DAP plates are not hypermutators indicates that these mutants emerged under CHX selection. It remains to be determined at what point in the CHX serial passage experiments reduced DAP susceptibility emerged, since only the beginning and endpoints of the evolution experiments were assessed in this study. Deep sequencing of populations at the beginning, middle, and end of the CHX passage experiments could be used in future studies to further examine the diversity and frequency of genetic variations arising as a result of serial subinhibitory CHX exposure. Moreover, it remains to be determined whether susceptibility to host-associated cationic antimicrobial peptides is also altered as a result of serial CHX exposure.

CHX, a cationic antiseptic, interacts with the bacterial cell membrane (12). Various mechanisms of reduced CHX susceptibility in Gram-negative and -positive bacteria have been reported. The two main mechanisms include CHX efflux (23, 35, 37, 38, 45–48) and changes in outer membrane content (49, 50). In this study, we have confirmed a role for the heterodimeric ABC transporter in CHX susceptibility in VREfm. Deletion of *efrEF* increases susceptibility of E. faecium 410 to CHX, and an amino acid substitution in EfrE is associated with decreased susceptibility to CHX. By lipidomic analysis, we found that the presence of two ethoxylated fatty amine compounds was abolished in the *efrEF* deletion mutant relative to the wild type. It remains to be determined whether and how these compounds are protective against CHX.

Daptomycin (DAP) is a cyclic lipopeptide antibiotic used to treat infections caused by multidrug-resistant Gram-positive pathogens, including VREfm (51–53). DAP resistance arises by mutation, leading to treatment failure (54). DAP is a negatively charged molecule that requires calcium ions for activity. Interaction of the cationic DAP-calcium complex with the membrane induces daptomycin oligomerization, membrane phospholipid remodeling, and other physiological alterations, ultimately leading to cell death (55–60). Broadly speaking, alterations in cell surface composition and in cellular stress responses are associated with reduced DAP susceptibility in Gram-positive bacteria (61, 62). In this study, we identified adaptive changes in genes with predicted or experimentally confirmed roles in chlorhexidine susceptibility (*efrE*), global nutritional stress response (*relA*), nucleotide metabolism (*cmk*), phosphate acquisition (*phoU*), and glycolipid biosynthesis (*bgsB*) occurring in the CHX-passaged mutants with reduced DAP susceptibilities. Of these, *relA* has been directly implicated in DAP resistance. Production of the alarmone (p)ppGpp is controlled by RelA. Changes in ppGpp concentration modulate the stringent stress response and impact antibiotic tolerance and virulence in Enterococcus (63, 64). Mutations in *relA* have previously been associated with *in vitro* (55) and *in vivo* (65) emergence of DAP resistance in Bacillus subtilis and E. faecium, respectively.

We identified other phenotypes occurring in CHX-evolved VREfm with reduced DAP susceptibility. The DAP-A1, DAP-A2, and DAP-B1 populations each had significantly lower growth rates than did the wild-type parent, indicating that CHX adaptation comes with a fitness cost. However, the growth rate of DAP-B1 was not significantly different than the parent strain. Moreover, significant changes in cellular membrane phospholipid and glycolipid content, particularly for CL, occurred in the DAP-A1 and DAP-A2 populations, but not the DAP-B populations. It is likely that these different phenotypes reflect the different genotypes of the CHX-evolved populations, since different mutations emerged in the two independent evolution experiments. A weakness of our study is that we did not assess each mutation by introducing them back to the parent strain in controlled genetic experiments, in order to assess the contribution of each to DAP susceptibility and cellular lipid content. This will be a focus of future work.

Our work has clinical implications. If subinhibitory CHX exposure selects for VREfm mutants with enhanced abilities to tolerate or resist DAP, these mutants could contribute to treatment failures with DAP. Frequent improper use of CHX (i.e., the presence of subinhibitory concentrations on patient skin) may favor the emergence and persistence of these VREfm mutants in health care settings. Surveillance of VREfm from hospital wards utilizing CHX bathing would be useful for monitoring the long-term impact of CHX bathing on these organisms. Routine subinhibitory CHX exposure may be a contributing factor to the clinical emergence of DAP resistance in VREfm.

## MATERIALS AND METHODS

### Bacterial strains and growth conditions.

The bacterial strains used in this study are listed in Table 1. E. faecium was cultured at 37°C on brain heart infusion (BHI) agar or in BHI broth without agitation unless otherwise stated. The CHX product used for experiments was Hibiclens (4% [wt/vol] chlorhexidine gluconate with 4% isopropyl alcohol). Chloramphenicol was used at 15 μg/ml for E. coli and E. faecium.

### Routine molecular biology techniques.

E. faecium genomic DNA was isolated according to a previously published protocol (66). DNA fragments were purified using the Purelink PCR purification kit (Thermo Fisher). *Taq* polymerase (New England BioLabs) was used for routine PCR. Routine DNA sequencing was performed by the Massachusetts General Hospital DNA core facility (Boston, MA). The primers used are shown in Table S1 in the supplemental material.

### MIC determinations.

Vancomycin, ampicillin, and CHX MICs were determined by broth microdilution in BHI as described previously (24). CHX MICs were recorded at 48 h postinoculation. DAP MICs were measured using Etest (bioMérieux, Inc.) strips on Mueller-Hinton agar (MHA) plates according to the manufacturer's instructions. MICs were independently assessed at least three times.

### RT-qPCR.

E. faecium 1,231,410 (E. faecium 410) and 1,141,733 cultures treated for 15 min with 0× (control) and 1× MIC CHX were harvested and mixed with 2 volumes of RNAprotect Bacteria Reagent (Qiagen) according to the manufacturer's recommendations and a previously published protocol (24). Then, 100 ng RNA was used to synthesize cDNA with Superscript II (Life Technologies), and 5 ng cDNA was used as the template in RT-qPCR with primers to amplify internal regions of *liaX* or *clpX*. Threshold cycle (*C_T_*) values were used to calculate the fold change of *liaX* gene expression (normalized by *clpX* expression) between 1× MIC CHX-treated cultures and control cultures (*n* = 2 independent trials).

### Serial passage experiments.

Wild-type E. faecium 410 was used for *in vitro* serial passage according to a previously published protocol (27). The CHX 1× MIC value for E. faecium 410 was 4.9 μg/ml on day 1. For serial passage, overnight culture was adjusted to an optical density at 600 nm (OD_600_) of 0.1 and exposed to CHX below, at, and above the MIC in BHI broth. The cultures were incubated at 37°C, and after 24 h, the cultures with visible growth in the highest drug concentration were used as an inoculum for the next round of passaging on day 2. Again, the inoculum OD_600_ was adjusted to 0.1, and cultures were exposed to CHX as described above. Passages were performed for 21 days. The serial passage experiment was performed independently twice, referred to as experiments A and B. Cultures from the 22nd day of experiment A (referred to as population A) and experiment B (referred to as population B) were analyzed in this study. E. faecium 410 cultures were also passaged in BHI without CHX for 21 days in two independent trials. Populations from day 21 of these trials are referred to as 410-P1 and 410-P2 in this study.

### Agar daptomycin susceptibility assay.

Cultures of populations A and B were inoculated directly from glycerol stocks into BHI broth and incubated overnight. Cultures were serially diluted in 1× phosphate-buffered saline (PBS) and spotted on MHA plates supplemented with calcium (50 μg/ml) with or without 10 μg/ml DAP. Plates were incubated for 24 to 36 h at 37°C prior to colony counting. Three (for population A) or four (for population B) independent trials were performed. A one-tailed Student *t* test was used to assess significance. E. faecium 410 unpassaged (seven trials) and 410-P1 and 410-P2 populations (three trials each) were assayed as controls. Colonies arising on DAP plates from two independent trials each for populations A and B were pooled, inoculated in BHI broth, and cryopreserved.

### Genome sequencing and analysis.

Genomic DNA was isolated from overnight broth cultures. The DAP MIC was confirmed for these cultures by an Etest assay. Library preparation and 2×150 paired-end Illumina sequencing was performed by Molecular Research LP (Shallowater, TX). The wild-type E. faecium 410 strain was sequenced as a control. Sequence reads were first assembled to the E. faecium 410 draft genome sequence (NZ_ACBA00000000.1) using default parameters for local alignment in CLC Genomics Workbench (Qiagen). Polymorphisms in the read assemblies were detected using the basic variant mapping tool for sites with ≥10-fold coverage. Variations occurring with ≥50% frequency were compared to the wild-type E. faecium 410 read assembly to find mutant-specific SNPs. To detect putative transposon hops, the read-mapping parameters were changed to global instead of local alignment, and regions of interest were manually analyzed. ≥98.8% of the nucleotide positions in the E. faecium 410 reference were covered ≥10-fold and included in variation analyses. Variants unique to the DAP-resistant mutants were confirmed by Sanger sequencing.

### Phosphate assay.

A commercially available phosphate colorimetric kit (Sigma-Aldrich; MAK030) and a previously published protocol (67) were utilized to measure intracellular inorganic phosphate (P_i_) levels in wild-type and DAP-B1 cultures. Overnight cultures were diluted to an OD_600_ of 0.01 in 50 ml of prewarmed BHI, followed by incubation at 37°C with shaking at 100 rpm. The phosphate levels were measured at four time points: 1, OD_600_ = 0.4 to 0.5; 2, OD_600_ = 0.5 to 0.6; 3, OD_600_ = 0.6 to 0.7; and 4, OD_600_ = 0.7 to 0.8. For each time point, 100-μl culture was serially diluted in 0.9% sterile NaCl and spotted on BHI plates for CFU determination. Also, 1 ml of the culture was incubated on ice for 5 min and then pelleted at 13,300 × *g* for 2 min at 4°C. The pellet was washed twice in 1 ml of double-distilled water, resuspended in 0.5 ml of double-distilled water, and then disrupted three times (Fast-Prep-24; MPBio) at 6.5 m/s for 30 s. The homogenized samples were centrifuged at 13,300 × *g* for 15 min at 4°C. Twenty-five μl of the supernatant was diluted with 25 μl double-distilled water, and the P_i_ levels were measured per the manufacturer's instructions. The phosphate levels were normalized by determining the CFU. A one-tailed Student *t* test was used to assess significance from three independent trials.

### Bacterial growth curves.

Overnight broth cultures inoculated from glycerol stocks were diluted to an OD_600_ of 0.01 in 50 ml of prewarmed BHI and incubated at 37°C with shaking at 100 rpm. The OD_600_ values were measured for 6 h. The experiment was performed independently three times, and a one-tailed Student *t* test was used to assess significance.

### Rifampin resistance frequency.

Overnight cultures of E. faecium 410 wild-type and DAP-resistant mutant strains were serially diluted in 1× PBS and spot plated on BHI agar to obtain the total CFU counts. Three milliliters of the cultures were pelleted and spread onto BHI agar plates supplemented with 50 μg/ml rifampin to obtain rifampin-resistant CFU counts. Plates were incubated for 24 to 48 h. Four independent trials were performed, and the significance value was calculated using a one-tailed Student *t* test.

### Gene deletion and agar CHX susceptibility assay.

Loci (EFTG_02287-02288) encoding the predicted ABC transport system EfrEF were deleted in-frame utilizing plasmid pHA101 in wild-type E. faecium 410 and the DAP-A2 mutant. Briefly, 999-bp flanking upstream and downstream regions of the genes of interest were amplified using the primers in Table S1 in the supplemental material, ligated with the plasmid pHA101, and propagated in E. coli EC1000. The construct, pPB301, was sequence verified and electroporated into E. faecium 410 according to a previously published protocol (24). Temperature shift at a nonpermissible temperature of 42°C and counterselection with *p*-chlorophenylalanine was monitored according to a previously published protocol (68). Deletion of EFTG_02287-02288 in the mutant strains (E. faecium PB301 and PB302) was confirmed by Sanger sequencing. Next, overnight cultures of wild-type E. faecium 410, PB301, DAP-A2, and PB302 strains were serially diluted and spot plated onto BHI plates supplemented with or without 1/8×, 1/4×, and 1/2× MIC CHX. The plates were incubated at 37°C for 24 to 36 h, and the CFU/ml values from three independent trials were quantified. A one-tailed Student *t* test was used to assess significance.

### Complementation assay for CHX susceptibility.

To complement the transporter deletion *in trans*, the genes EFTG_02287-02288 with their putative native promoter were amplified from wild-type E. faecium 410 or the DAP-A2 mutant and ligated with pLZ12 (69) to generate constructs pAH201 and pPB202. The complementation constructs were sequence verified and electroporated into the *efrEF* deletion mutant (PB301) to generate strains AH102 and PB303. The empty pLZ12 plasmid was also transformed into the deletion mutant as a control (strain AH101). To assay complementation, overnight cultures were serially diluted and spot plated on BHI-chloramphenicol (15 μg/ml) plates supplemented with or without 1/8×, 1/4×, and 1/2× MIC CHX. The plates were incubated at 37°C for 24 to 36 h. The CFU/ml values for three independent trials were quantified, and a one-tailed Student *t* test was used to assess significance.

### Lipidomic analysis.

Cultures were inoculated from glycerol stock into 10 ml of BHI broth, followed by incubation at 37°C overnight. The 10-ml cultures were added to prewarmed 250 ml of BHI and incubated until reaching an OD_600_ of ∼0.6. Then, 100 μl was removed for DAP Etest testing on MHA plates. Also, 15 ml of the cultures was added to 2 volumes of RNAprotect Bacteria Reagent according to the manufacturer's recommendations and a previously published protocol (24), and samples were used to assess *cls*, *cmk*, *clpX*, or *liaX* expression by RT-qPCR as described above. The remaining culture was pelleted at 10,000 rpm at 4°C. Cell pellets were stored at −80°C prior to lipid extraction by the Bligh and Dyer method (70). Lipid analysis by normal-phase liquid chromatography ESI/MS (LC-ESI/MS) was performed using an Agilent 1200 Quaternary LC system coupled to a high-resolution TripleTOF5600 mass spectrometer (Sciex, Framingham, MA), as previously described (71, 72). Normal-phase LC was performed on an Agilent 1200 Quaternary LC system using an Ascentis Silica HPLC column, (5 μm; 25 cm by 2.1 mm; Sigma-Aldrich). Mobile phase A and B solvents, flow conditions, and instrumental settings for ESI/MS and MS/MS were as previously published (73). Data analysis was performed using Analyst TF1.5 software (Sciex). To compare wild-type and DAP mutant strains, three independent lipidomics trials were performed. To compare wild-type and Δ*efrEF* strains, two independent trials were performed.

### Accession number(s).

Raw Illumina sequence reads generated in this study are available in the Sequence Read Archive under accession number SRP108331.

## Supplementary Material

Supplemental material

## References

[1] Agudelo Higuita NI, Huycke MM 2014 Enterococcal disease, epidemiology, and implications for treatment, p 45–70. *In* Gilmore MS, Clewell DB, Ike Y, Shankar N (ed), Enterococci: from commensals to leading causes of drug-resistant infection. Massachusetts Eye and Ear Infirmary, Boston, MA.

[2] Arias CA, Murray BE 2012 The rise of the *Enterococcus*: beyond vancomycin resistance. Nat Rev Microbiol 10:266–278. doi:10.1038/nrmicro2761. 22421879 PMC3621121

[3] Gilmore MS, Lebreton F, van Schaik W 2013 Genomic transition of enterococci from gut commensals to leading causes of multidrug-resistant hospital infection in the antibiotic era. Curr Opin Microbiol 16:10–16. doi:10.1016/j.mib.2013.01.006. 23395351 PMC3649759

[4] Sievert DM, Ricks P, Edwards JR, Schneider A, Patel J, Srinivasan A, Kallen A, Limbago B, Fridkin S, National Healthcare Safety Network. 2013 Antimicrobial-resistant pathogens associated with healthcare-associated infections: summary of data reported to the National Healthcare Safety Network at the Centers for Disease Control and Prevention, 2009-2010. Infect Control Hosp Epidemiol 34:1–14. doi:10.1086/668770. 23221186

[5] Centers for Disease Control and Prevention. 2011 Vital signs: central line-associated blood stream infections–United States, 2001, 2008, and 2009. MMWR Morb Mortal Wkly Rep 60:243–248. 21368740

[6] Muto CA, Jernigan JA, Ostrowsky BE, Richet HM, Jarvis WR, Boyce JM, Farr BM, Shea. 2003 SHEA guideline for preventing nosocomial transmission of multidrug-resistant strains of *Staphylococcus aureus* and enterococcus. Infect Control Hosp Epidemiol 24:362–386. doi:10.1017/S0195941700083375. 12785411

[7] Boyce JM 2007 Environmental contamination makes an important contribution to hospital infection. J Hosp Infect 65(Suppl 2):S50–S54. doi:10.1016/S0195-6701(07)60015-2. 17540242

[8] Weber DJ, Anderson D, Rutala WA 2013 The role of the surface environment in healthcare-associated infections. Curr Opin Infect Dis 26:338–344. doi:10.1097/QCO.0b013e3283630f04. 23743816

[9] Hota B 2004 Contamination, disinfection, and cross-colonization: are hospital surfaces reservoirs for nosocomial infection? Clin Infect Dis 39:1182–1189. doi:10.1086/424667. 15486843 PMC7107941

[10] Davies A 1973 The mode of action of chlorhexidine. J Periodont Res Suppl 12:68–75. doi:10.1111/j.1600-0765.1973.tb02167.x. 4269603

[11] Davies GE, Francis J, Martin AR, Rose FL, Swain G 1954 1:6-Di-4′-chlorophenyldiguanidohexane (hibitane): laboratory investigation of a new antibacterial agent of high potency. Br J Pharmacol Chemother 9:192–196. doi:10.1111/j.1476-5381.1954.tb00840.x. 13172429 PMC1509439

[12] Koontongkaew S, Jitpukdeebodintra S 1995 Interaction of chlorhexidine with cytoplasmic membranes of *Streptococcus mutans* GS-5. Caries Res 29:413–417. doi:10.1159/000262101. 8521445

[13] Hugo WB, Longworth AR 1966 The effect of chlorhexidine on the electrophoretic mobility, cytoplasmic constituents, dehydrogenase activity, and cell walls of *Escherichia coli* and *Staphylococcus aureus*. J Pharm Pharmacol 18:569–578. doi:10.1111/j.2042-7158.1966.tb07935.x. 4381940

[14] Hugo WB, Longworth AR 1964 Some aspects of the mode of action of chlorhexidine. J Pharm Pharmacol 16:655–662. doi:10.1111/j.2042-7158.1964.tb07384.x. 14226440

[15] Hugo WB, Longworth AR 1965 Cytological aspects of the mode of action of chlorhexidine diacetate. J Pharm Pharmacol 17:28–32. doi:10.1111/j.2042-7158.1965.tb07562.x. 14285692

[16] Marschall J, Mermel LA, Fakih M, Hadaway L, Kallen A, O'Grady NP, Pettis AM, Rupp ME, Sandora T, Maragakis LL, Yokoe DS, Society for Healthcare Epidemiology of America. 2014 Strategies to prevent central line-associated bloodstream infections in acute care hospitals: 2014 update. Infect Control Hosp Epidemiol 35:753–771. doi:10.1086/676533. 24915204

[17] Supple L, Kumaraswami M, Kundrapu S, Sunkesula V, Cadnum JL, Nerandzic MM, Tomas M, Donskey CJ 2015 Chlorhexidine only works if applied correctly: use of a simple colorimetric assay to provide monitoring and feedback on effectiveness of chlorhexidine application. Infect Control Hosp Epidemiol 36:1095–1097. doi:10.1017/ice.2015.124. 26074153

[18] Rupp ME, Lisco SJ, Lipsett PA, Perl TM, Keating K, Civetta JM, Mermel LA, Lee D, Dellinger EP, Donahoe M, Giles D, Pfaller MA, Maki DG, Sherertz R 2005 Effect of a second-generation venous catheter impregnated with chlorhexidine and silver sulfadiazine on central catheter-related infections: a randomized, controlled trial. Ann Intern Med 143:570–580. doi:10.7326/0003-4819-143-8-200510180-00007. 16230723

[19] Noto MJ, Wheeler AP 2015 Understanding chlorhexidine decolonization strategies. Intensive Care Med 41:1351–1354. doi:10.1007/s00134-015-3846-6. 26088910

[20] Noto MJ, Domenico HJ, Byrne DW, Talbot T, Rice TW, Bernard GR, Wheeler AP 2015 Chlorhexidine bathing and health care-associated infections: a randomized clinical trial. JAMA 313:369–378. doi:10.1001/jama.2014.18400. 25602496 PMC4383133

[21] Pittet D, Angus DC 2015 Daily chlorhexidine bathing for critically ill patients: a note of caution. JAMA 313:365–366. doi:10.1001/jama.2014.18482. 25603492

[22] Popovich KJ, Lyles R, Hayes R, Hota B, Trick W, Weinstein RA, Hayden MK 2012 Relationship between chlorhexidine gluconate skin concentration and microbial density on the skin of critically ill patients bathed daily with chlorhexidine gluconate. Infect Control Hosp Epidemiol 33:889–896. doi:10.1086/667371. 22869262 PMC3632447

[23] Wand ME, Bock LJ, Bonney LC, Sutton JM 2017 Mechanisms of increased resistance to chlorhexidine and cross-resistance to colistin following exposure of *Klebsiella pneumoniae* clinical isolates to chlorhexidine. Antimicrob Agents Chemother 61:e01162-16. doi:10.1128/AAC.01162-16. 27799211 PMC5192135

[24] Bhardwaj P, Ziegler E, Palmer KL 2016 Chlorhexidine induces VanA-type vancomycin resistance genes in enterococci. Antimicrob Agents Chemother 60:2209–2221. doi:10.1128/AAC.02595-15. 26810654 PMC4808233

[25] Palmer KL, Gilmore MS 2010 Multidrug-resistant enterococci lack CRISPR-cas. mBio 1:e00227-10. doi:10.1128/mBio.00227-10. 21060735 PMC2975353

[26] Palmer KL, Godfrey P, Griggs A, Kos VN, Zucker J, Desjardins C, Cerqueira G, Gevers D, Walker S, Wortman J, Feldgarden M, Haas B, Birren B, Gilmore MS 2012 Comparative genomics of enterococci: variation in *Enterococcus faecalis*, clade structure in *E. faecium*, and defining characteristics of *E. gallinarum* and *E. casseliflavus*. mBio 3:e00318-11. doi:10.1128/mBio.00318-11. 22354958 PMC3374389

[27] Palmer KL, Daniel A, Hardy C, Silverman J, Gilmore MS 2011 Genetic basis for daptomycin resistance in enterococci. Antimicrob Agents Chemother 55:3345–3356. doi:10.1128/AAC.00207-11. 21502617 PMC3122436

[28] Arias CA, Panesso D, McGrath DM, Qin X, Mojica MF, Miller C, Diaz L, Tran TT, Rincon S, Barbu EM, Reyes J, Roh JH, Lobos E, Sodergren E, Pasqualini R, Arap W, Quinn JP, Shamoo Y, Murray BE, Weinstock GM 2011 Genetic basis for in vivo daptomycin resistance in enterococci. N Engl J Med 365:892–900. doi:10.1056/NEJMoa1011138. 21899450 PMC3205971

[29] Davlieva M, Shi Y, Leonard PG, Johnson TA, Zianni MR, Arias CA, Ladbury JE, Shamoo Y 2015 A variable DNA recognition site organization establishes the LiaR-mediated cell envelope stress response of enterococci to daptomycin. Nucleic Acids Res 43:4758–4773. doi:10.1093/nar/gkv321. 25897118 PMC4482077

[30] Davlieva M, Tovar-Yanez A, DeBruler K, Leonard PG, Zianni MR, Arias CA, Shamoo Y 2016 An adaptive mutation in *Enterococcus faecium* LiaR associated with antimicrobial peptide resistance mimics phosphorylation and stabilizes LiaR in an activated state. J Mol Biol 428:4503–4519. doi:10.1016/j.jmb.2016.09.016. 27670715 PMC5085866

[31] Diaz L, Tran TT, Munita JM, Miller WR, Rincon S, Carvajal LP, Wollam A, Reyes J, Panesso D, Rojas NL, Shamoo Y, Murray BE, Weinstock GM, Arias CA 2014 Whole-genome analyses of *Enterococcus faecium* isolates with diverse daptomycin MICs. Antimicrob Agents Chemother 58:4527–4534. doi:10.1128/AAC.02686-14. 24867964 PMC4136017

[32] Miller C, Kong J, Tran TT, Arias CA, Saxer G, Shamoo Y 2013 Adaptation of *Enterococcus faecalis* to daptomycin reveals an ordered progression to resistance. Antimicrob Agents Chemother 57:5373–5383. doi:10.1128/AAC.01473-13. 23959318 PMC3811304

[33] Clinical and Laboratory Standards Institute. 2017 Performance standards for antimicrobial susceptibility testing; 27th ed Supplement M100. CLSI, Wayne, PA.

[34] Guzman Prieto AM, Wijngaarden J, Braat JC, Rogers MR, Majoor E, Brouwer EC, Zhang X, Bayjanov JR, Bonten MJ, Willems RJ, van Schaik W 2017 The two-component system ChtRS contributes to chlorhexidine tolerance in *Enterococcus faecium*. Antimicrob Agents Chemother 61:e02122-16. doi:10.1128/AAC.02122-16. 28242664 PMC5404517

[35] Hurlimann LM, Corradi V, Hohl M, Bloemberg GV, Tieleman DP, Seeger MA 2016 The heterodimeric ABC transporter EfrCD mediates multidrug efflux in *Enterococcus faecalis*. Antimicrob Agents Chemother 60:5400–5411. doi:10.1128/AAC.00661-16. 27381387 PMC4997860

[36] Krogh A, Larsson B, von Heijne G, Sonnhammer EL 2001 Predicting transmembrane protein topology with a hidden Markov model: application to complete genomes. J Mol Biol 305:567–580. doi:10.1006/jmbi.2000.4315. 11152613

[37] Davis DR, McAlpine JB, Pazoles CJ, Talbot MK, Alder EA, White C, Jonas BM, Murray BE, Weinstock GM, Rogers BL 2001 *Enterococcus faecalis* multi-drug resistance transporters: application for antibiotic discovery. J Mol Microbiol Biotechnol 3:179–184. 11321571

[38] Davis DV, Rogers BL, White AC 10 2001 Multidrug resistance (mdr) efflux pump polypeptides. Google Patent WO 2001079257 A2.

[39] Mechold U, Murphy H, Brown L, Cashel M 2002 Intramolecular regulation of the opposing (p)ppGpp catalytic activities of Rel(Seq), the Rel/Spo enzyme from *Streptococcus equisimilis*. J Bacteriol 184:2878–2888. doi:10.1128/JB.184.11.2878-2888.2002. 12003927 PMC135074

[40] Hogg T, Mechold U, Malke H, Cashel M, Hilgenfeld R 2004 Conformational antagonism between opposing active sites in a bifunctional RelA/SpoT homolog modulates (p)ppGpp metabolism during the stringent response. Cell 117:57–68. doi:10.1016/S0092-8674(04)00260-0. 15066282

[41] Rice P, Longden I, Bleasby A 2000 EMBOSS: the European molecular biology open software suite. Trends Genet 16:276–277. doi:10.1016/S0168-9525(00)02024-2. 10827456

[42] Theilacker C, Sava I, Sanchez-Carballo P, Bao Y, Kropec A, Grohmann E, Holst O, Huebner J 2011 Deletion of the glycosyltransferase bgsB of *Enterococcus faecalis* leads to a complete loss of glycolipids from the cell membrane and to impaired biofilm formation. BMC Microbiol 11:67. doi:10.1186/1471-2180-11-67. 21470413 PMC3083329

[43] Theilacker C, Sanchez-Carballo P, Toma I, Fabretti F, Sava I, Kropec A, Holst O, Huebner J 2009 Glycolipids are involved in biofilm accumulation and prolonged bacteraemia in *Enterococcus faecalis*. Mol Microbiol 71:1055–1069. doi:10.1111/j.1365-2958.2008.06587.x. 19170884

[44] Gonzalez-Rodriguez MV, Dopico-Garcia MS, Noguerol-Cal R, Carballeira-Amarelo T, Lopez-Vilarino JM, Fernandez-Martinez G 2010 Application of liquid chromatography in polymer nonionic antistatic additives analysis. J Sep Sci 33:3595–3603. doi:10.1002/jssc.201000460. 20931612

[45] Russell AD 2002 Introduction of biocides into clinical practice and the impact on antibiotic-resistant bacteria. Symp Ser Soc Appl Microbiol 2002:121S–135S. doi:10.1046/j.1365-2672.92.5s1.12.x. 12481837

[46] Costa SS, Viveiros M, Amaral L, Couto I 2013 Multidrug efflux pumps in *Staphylococcus aureus*: an update. Open Microbiol J 7:59–71. doi:10.2174/1874285801307010059. 23569469 PMC3617543

[47] Rajamohan G, Srinivasan VB, Gebreyes WA 2010 Novel role of *Acinetobacter baumannii* RND efflux transporters in mediating decreased susceptibility to biocides. J Antimicrob Chemother 65:228–232. doi:10.1093/jac/dkp427. 20008046

[48] Hassan KA, Jackson SM, Penesyan A, Patching SG, Tetu SG, Eijkelkamp BA, Brown MH, Henderson PJ, Paulsen IT 2013 Transcriptomic and biochemical analyses identify a family of chlorhexidine efflux proteins. Proc Natl Acad Sci U S A 110:20254–20259. doi:10.1073/pnas.1317052110. 24277845 PMC3864336

[49] Tattawasart U, Maillard JY, Furr JR, Russell AD 2000 Outer membrane changes in *Pseudomonas stutzeri* resistant to chlorhexidine diacetate and cetylpyridinium chloride. Int J Antimicrob Agents 16:233–238. doi:10.1016/S0924-8579(00)00206-5. 11091041

[50] Westergren G, Emilson CG 1980 In vitro development of chlorhexidine resistance in *Streptococcus sanguis* and its transmissibility by genetic transformation. Scand J Dent Res 88:236–243. 6932090 10.1111/j.1600-0722.1980.tb01220.x

[51] Tally FP, Zeckel M, Wasilewski MM, Carini C, Berman CL, Drusano GL, Oleson FB Jr 1999 Daptomycin: a novel agent for Gram-positive infections. Expert Opin Invest Drugs 8:1223–1238. doi:10.1517/13543784.8.8.1223. 15992147

[52] Akins RL, Rybak MJ 2001 Bactericidal activities of two daptomycin regimens against clinical strains of glycopeptide intermediate-resistant *Staphylococcus aureus*, vancomycin-resistant *Enterococcus faecium*, and methicillin-resistant *Staphylococcus aureus* isolates in an in vitro pharmacodynamic model with simulated endocardial vegetations. Antimicrob Agents Chemother 45:454–459. doi:10.1128/AAC.45.2.454-459.2001. 11158740 PMC90312

[53] Alder JD 2005 Daptomycin: a new drug class for the treatment of Gram-positive infections. Drugs Today (Barc) 41:81–90. doi:10.1358/dot.2005.41.2.882660. 15821781

[54] Munita JM, Murray BE, Arias CA 2014 Daptomycin for the treatment of bacteraemia due to vancomycin-resistant enterococci. Int J Antimicrob Agents 44:387–395. doi:10.1016/j.ijantimicag.2014.08.002. 25261158 PMC4417356

[55] Hachmann AB, Sevim E, Gaballa A, Popham DL, Antelmann H, Helmann JD 2011 Reduction in membrane phosphatidylglycerol content leads to daptomycin resistance in *Bacillus subtilis*. Antimicrob Agents Chemother 55:4326–4337. doi:10.1128/AAC.01819-10. 21709092 PMC3165287

[56] Muraih JK, Pearson A, Silverman J, Palmer M 2011 Oligomerization of daptomycin on membranes. Biochim Biophys Acta 1808:1154–1160. doi:10.1016/j.bbamem.2011.01.001. 21223947

[57] Jung D, Rozek A, Okon M, Hancock RE 2004 Structural transitions as determinants of the action of the calcium-dependent antibiotic daptomycin. Chem Biol 11:949–957. doi:10.1016/j.chembiol.2004.04.020. 15271353

[58] Ho SW, Jung D, Calhoun JR, Lear JD, Okon M, Scott WR, Hancock RE, Straus SK 2008 Effect of divalent cations on the structure of the antibiotic daptomycin. Eur Biophys J 37:421–433. doi:10.1007/s00249-007-0227-2. 17968536

[59] Straus SK, Hancock RE 2006 Mode of action of the new antibiotic for Gram-positive pathogens daptomycin: comparison with cationic antimicrobial peptides and lipopeptides. Biochim Biophys Acta 1758:1215–1223. doi:10.1016/j.bbamem.2006.02.009. 16615993

[60] Muller A, Wenzel M, Strahl H, Grein F, Saaki TN, Kohl B, Siersma T, Bandow JE, Sahl HG, Schneider T, Hamoen LW 2016 Daptomycin inhibits cell envelope synthesis by interfering with fluid membrane microdomains. Proc Natl Acad Sci U S A 113:E7077–E7086. doi:10.1073/pnas.1611173113. PMC5111643 27791134

[61] Bayer AS, Schneider T, Sahl HG 2013 Mechanisms of daptomycin resistance in *Staphylococcus aureus*: role of the cell membrane and cell wall. Ann N Y Acad Sci 1277:139–158. doi:10.1111/j.1749-6632.2012.06819.x. 23215859 PMC3556211

[62] Tran TT, Munita JM, Arias CA 2015 Mechanisms of drug resistance: daptomycin resistance. Ann N Y Acad Sci 1354:32–53. doi:10.1111/nyas.12948. 26495887 PMC4966536

[63] Abranches J, Martinez AR, Kajfasz JK, Chavez V, Garsin DA, Lemos JA 2009 The molecular alarmone (p)ppGpp mediates stress responses, vancomycin tolerance, and virulence in *Enterococcus faecalis*. J Bacteriol 191:2248–2256. doi:10.1128/JB.01726-08. 19168608 PMC2655485

[64] Gaca AO, Kajfasz JK, Miller JH, Liu K, Wang JD, Abranches J, Lemos JA 2013 Basal levels of (p)ppGpp in *Enterococcus faecalis*: the magic beyond the stringent response. mBio 4:e00646-13. doi:10.1128/mBio.00646-13. 24065631 PMC3781836

[65] Honsa ES, Cooper VS, Mhaissen MN, Frank M, Shaker J, Iverson A, Rubnitz J, Hayden RT, Lee RE, Rock CO, Tuomanen EI, Wolf J, Rosch JW 2017 RelA mutant *Enterococcus faecium* with multiantibiotic tolerance arising in an immunocompromised host. mBio 8:e00066-17. doi:10.1128/mBio.02124-16. 28049149 PMC5210501

[66] Adams HM, Li X, Mascio C, Chesnel L, Palmer KL 2015 Mutations associated with reduced surotomycin susceptibility in *Clostridium difficile* and *Enterococcus* species. Antimicrob Agents Chemother 59:4139–4147. doi:10.1128/AAC.00526-15. 25941217 PMC4468737

[67] Mechler L, Herbig A, Paprotka K, Fraunholz M, Nieselt K, Bertram R 2015 A novel point mutation promotes growth phase-dependent daptomycin tolerance in *Staphylococcus aureus*. Antimicrob Agents Chemother 59:5366–5376. doi:10.1128/AAC.00643-15. 26100694 PMC4538524

[68] Thurlow LR, Thomas VC, Hancock LE 2009 Capsular polysaccharide production in *Enterococcus faecalis* and contribution of CpsF to capsule serospecificity. J Bacteriol 191:6203–6210. doi:10.1128/JB.00592-09. 19684130 PMC2753019

[69] Perez-Casal J, Caparon MG, Scott JR 1991 Mry, a trans-acting positive regulator of the M protein gene of *Streptococcus pyogenes* with similarity to the receptor proteins of two-component regulatory systems. J Bacteriol 173:2617–2624. doi:10.1128/jb.173.8.2617-2624.1991. 1849511 PMC207828

[70] Bligh EG, Dyer WJ 1959 A rapid method of total lipid extraction and purification. Can J Biochem Physiol 37:911–917. doi:10.1139/y59-099. 13671378

[71] Tan BK, Bogdanov M, Zhao J, Dowhan W, Raetz CR, Guan Z 2012 Discovery of a cardiolipin synthase utilizing phosphatidylethanolamine and phosphatidylglycerol as substrates. Proc Natl Acad Sci U S A 109:16504–16509. doi:10.1073/pnas.1212797109. 22988102 PMC3478633

[72] Li C, Tan BK, Zhao J, Guan Z 2016 In vivo and in vitro synthesis of phosphatidylglycerol by an *Escherichia coli* cardiolipin synthase. J Biol Chem 291:25144–25153. doi:10.1074/jbc.M116.762070. 27760827 PMC5122781

[73] Adams HM, Joyce LR, Guan Z, Akins RL, Palmer KL 2017 *Streptococcus mitis* and *S. oralis* lack a requirement for CdsA, the enzyme required for synthesis of major membrane phospholipids in bacteria. Antimicrob Agents Chemother 61:e02552-16. doi:10.1128/AAC.02552-16. 28223392 PMC5404519

[74] Leenhouts K, Buist G, Bolhuis A, ten Berge A, Kiel J, Mierau I, Dabrowska M, Venema G, Kok J 1996 A general system for generating unlabelled gene replacements in bacterial chromosomes. Mol Gen Genet 253:217–224. doi:10.1007/s004380050315. 9003306

