# Supplementary material for: Reduced Chlorhexidine and Daptomycin Susceptibility in Vancomycin-Resistant Enterococcus faecium after Serial Chlorhexidine Exposure
Source: Antimicrob Agents Chemother. 2017 Dec 21;62(1):e01235-17. doi: 10.1128/AAC.01235-17 (PMC5740357; doi:10.1128/AAC.01235-17)
Supplement: Supplemental material [file supp_62_1_e01235-17__index.html]

Reduced Chlorhexidine and Daptomycin Susceptibility in Vancomycin-Resistant Enterococcus faecium after Serial Chlorhexidine Exposure — Supplemental material 

# Reduced Chlorhexidine and Daptomycin Susceptibility in Vancomycin-Resistant Enterococcus faecium after Serial Chlorhexidine Exposure

## Supplemental material

- Supplemental file 1 -

  Figures S1 to S6 and Tables S1 and S2

  PDF, 2.0M
- Supplemental file 2 -

  Data Set S1

  XLSX, 49K
- Supplemental file 3 -

  Data Set S2

  XLSX, 13K
