# Supplementary material for: Reduced Chlorhexidine and Daptomycin Susceptibility in Vancomycin-Resistant Enterococcus faecium after Serial Chlorhexidine Exposure
Source: Antimicrob Agents Chemother. 2017 Dec 21;62(1):e01235-17. doi: 10.1128/AAC.01235-17 (PMC5740357; doi:10.1128/AAC.01235-17)
Supplement: Supplemental material [file AAC.01235-17_zac001186763s1.pdf]

Supplemental Figures and Tables

Figure S1. CHX induces *liaX* gene expression in *E. faecium*. RT-qPCR was used to quantify the expression of *liaX* upon exposure to CHX for 15 min as compared to control untreated condition. Expression of *liaX* was internally normalized to *clpX*, and expression in control cultures was set to 1 (not shown). The fold change in *liaX* expression in cultures treated with 1X MIC CHX relative to the control was quantified for two independent (Trial 1 and 2) experiments. Efm 410, *E. faecium* 1,231,410; Efm 733, *E. faecium* 1,141,733.

Figure S2. Quantification of genes expression levels in DAP mutants by RT-qPCR. RT-qPCR was used to quantify the expression of **A)** *liaX*, **B)** *chtR* and *chtS*, and **C)** *cls-1* and *cls-2* genes at exponential phase (OD_{600}~0.6) in DAP mutants compared to *E. faecium* 410. Expression of genes of interest was internally normalized to *clpX*, and expression in *E. faecium* 410 cultures was set to 1. The standard deviation was calculated from n=3 independent experiments and one-tailed Student's *t*-test was used to calculate significance value. *, $P < 0.05$. 410 wild-type, *E. faecium* 1,231,410.

Figure S3. Quantification of expression levels of cytidylate kinase (*cmk*) in DAP mutants. **A)** Gel electrophoresis analysis of *cmk* promoter amplicons in *E. faecium* 410 wild-type and DAP-A mutants. 100 ng of purified PCR products were analyzed for each on a 1% TAE gel. Presence of wild-type and IS*1251* insertion PCR bands in the *cmk* promoter were observed in the DAP-A mutants. **B)** RT-qPCR confirmed significant down-regulation of *cmk* transcript levels in the DAP mutants vs. *E. faecium* 410 during exponential growth (OD_{600}~0.6) in three independent trials. Expression of *cmk* was internally normalized to *clpX* and expression of *E. faecium* 410 was set to 1 (not shown). *, $P < 0.05$. 410 wt, *E. faecium* 1,231,410; Neg., negative control.

Figure S4. Quantification of intracellular organic phosphate (Pi) levels in *E. faecium* 410 wild-type and DAP-B1 mutant. Intracellular Pi levels were measured for wild-type and DAP strain DAP-B1 at different growth time points (OD_{600} 0.4-0.8) as described in materials and methods. The levels (pmoles) were normalized using CFU count. Standard deviation was calculated from n=3 independent experiments and significance value was calculated using one-tailed Student's *t* test. Time points: 1, OD_{600} 0.4-0.5; 2, OD_{600} 0.5-0.6; 3, OD_{600} 0.6-0.7 and 4, OD_{600} 0.7-0.8. *, $P < 0.05$. 410 wt, *E. faecium* 1,231,410.

Figure S5. Cardiolipin levels are decreased in DAP-A1 and DAP-A2 mutants. Lipidomic analysis of *E. faecium* 410 and DAP strains DAP-A1 and -A2 was performed by normal-phase LC-ESI/MS in the negative ion mode. The major lipids detected are phosphatidylglycerol (PG), cardiolipin (CL), diacylglycerol (DAG), monohexosyldiacylglycerol (MHDAG), dihexosyldiacylglycerol (DHDAG), phosphatidic acid (PA), and lysylphosphaditylglycerol (LPG). Shown are the total ion chromatograms (TIC) and the selected mass spectra of $[M-H]^-$ ion species of PG and CL from the first trial.

Figure S6. Lipid-like compounds (Atmer-163) are detected in *E. faecium* 410 but not in Δ*efrEF* mutant. A) Positive ion ESI mass spectra showing the detection of Atmer-163 (C_{13}) ($[M+H]^+$ at *m/z* 288) and Atmer-163 (C_{15}) ($[M+H]^+$ ion at *m/z* 316) in *E. faecium* 410, and their absence in the Δ*efrEF* mutant. **B**) Chemical structures and molecular formulae of Atmer-163 (C_{13}) and Atmer-163 (C_{15}). **C**) MS/MS spectrum of Atmer-163 (C_{15}) $[M+H]^+$ ion at *m/z* 316. The fragment ion structures are depicted.

Table S1. List of primers used in the study.

Table S2. Doubling time and rifampin mutation frequency for the DAP strains vs. *E. faecium* 410 wild-type.

Figure S1

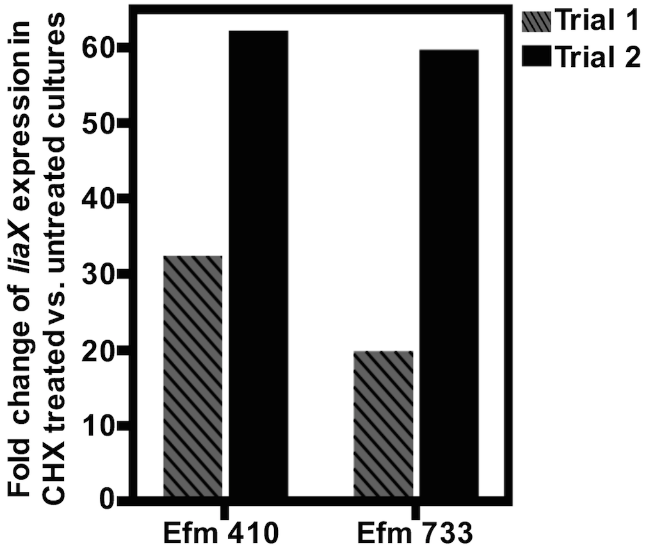

Fold change of *liaX* expression in
CHX treated vs. untreated cultures
60
50
40
30
20
10
0
Efm 410
Efm 733
Trial 1
Trial 2

Figure S2

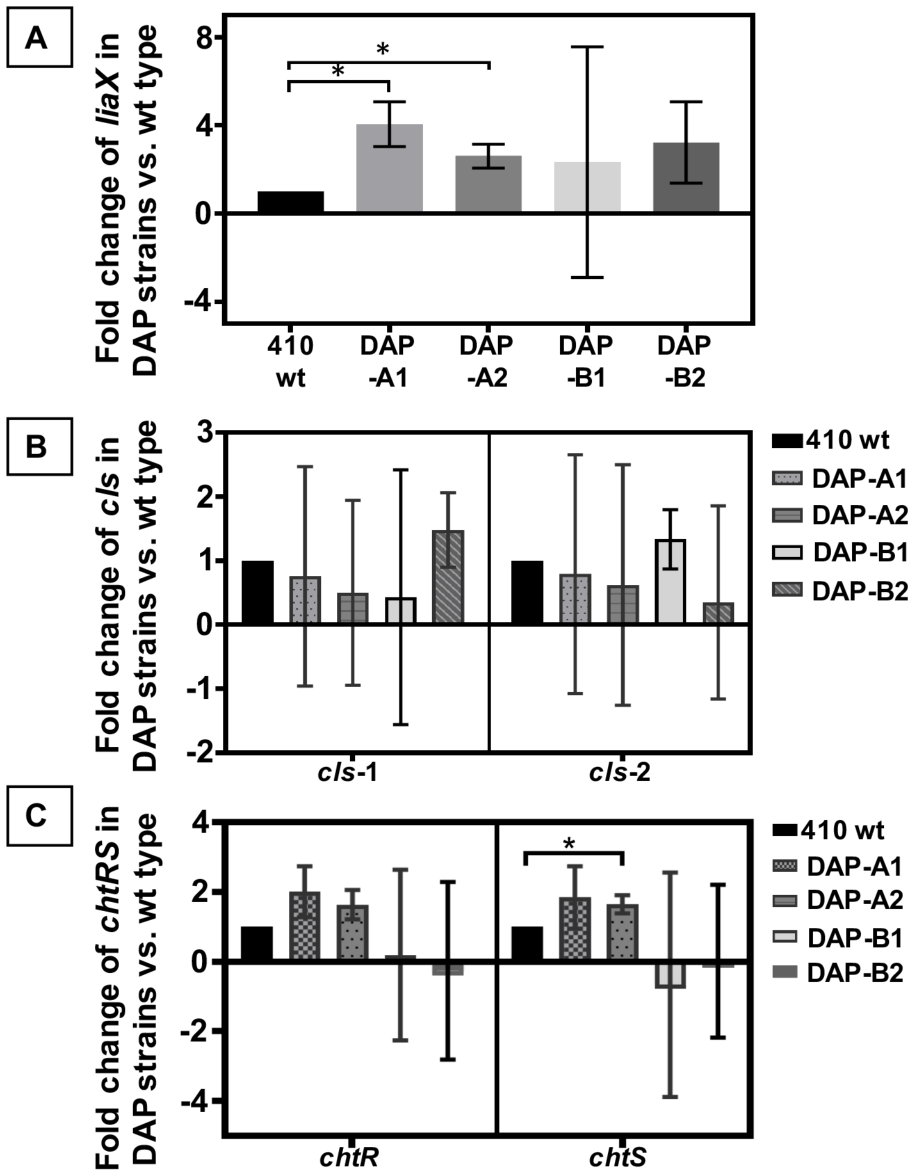
A
Fold change of liaX in DAP strains vs. wt type
8
4
0
-4
*
*
410 wt
DAP -A1
DAP -A2
DAP -B1
DAP -B2
B
Fold change of cls in DAP strains vs. wt type
3
2
1
0
-1
-2
cls-1
cls-2
410 wt
DAP-A1
DAP-A2
DAP-B1
DAP-B2
C
Fold change of chtRS in DAP strains vs. wt type
4
2
0
-2
-4
*
chtR
chtS
410 wt
DAP-A1
DAP-A2
DAP-B1
DAP-B2

Figure S3

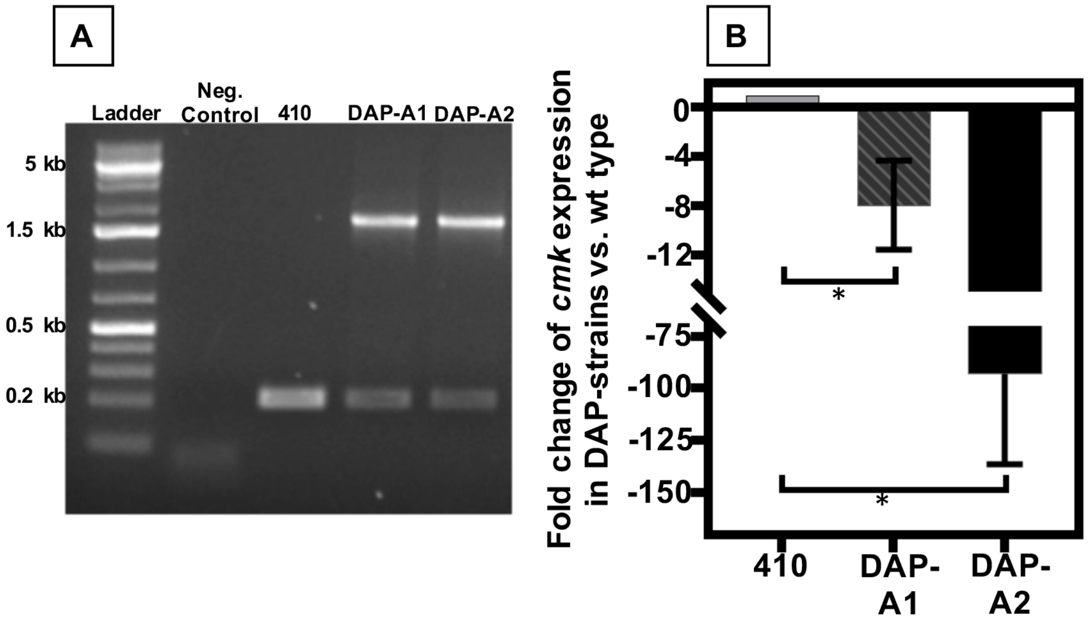
A
Ladder
Neg. Control
410
DAP-A1
DAP-A2
5 kb
1.5 kb
0.5 kb
0.2 kb
B
Fold change of cmk expression in DAP-strains vs. wt type
0
-4
-8
-12
-75
-100
-125
-150
*
*
410
DAP-A1
DAP-A2

Figure S4

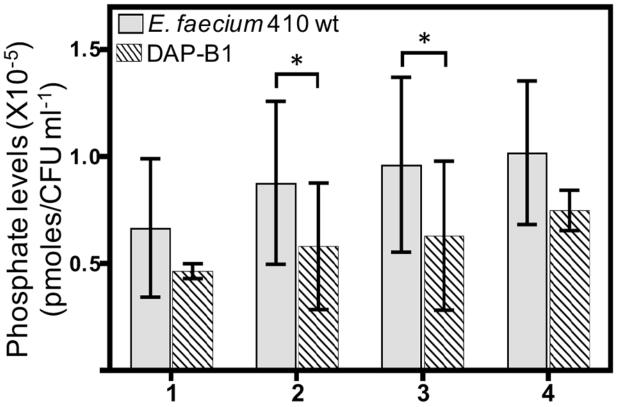

E. faecium 410 wt
DAP-B1
Phosphate levels (X10-5) (pmoles/CFU ml-1)
1.5
1.0
0.5
*
*
1
2
3
4

Figure S5

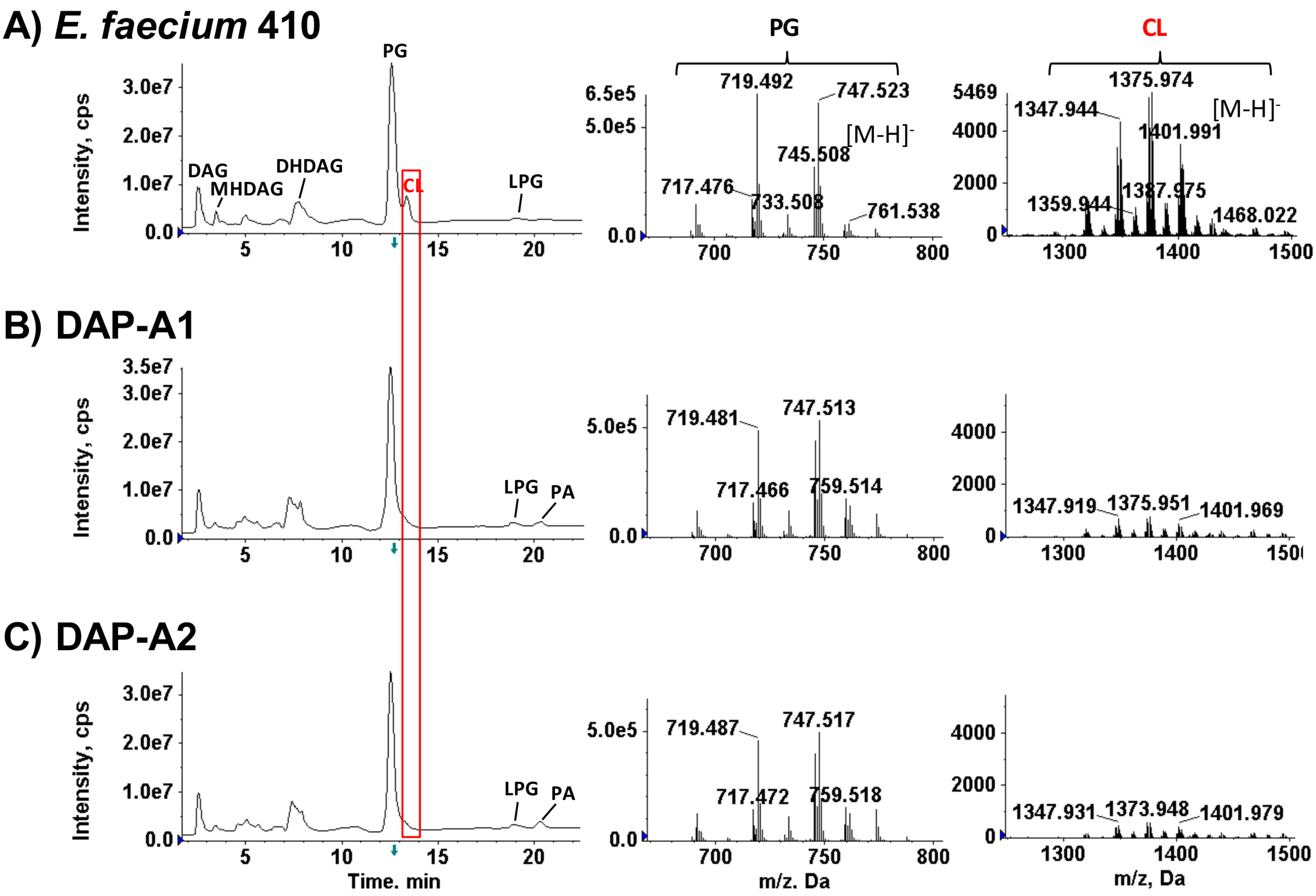

A) E. faecium 410
B) DAP-A1
C) DAP-A2
PG
CL
DAG
MHDAG
DHDAG
LPG
PA
Intensity, cps
Time. min
m/z. Da
m/z, Da
[M-H]-
719.492
747.523
745.508
717.476
733.508
761.538
1375.974
1347.944
1401.991
1359.944
1387.975
1468.022
719.481
747.513
717.466
759.514
1347.919
1375.951
1401.969
719.487
747.517
717.472
759.518
1347.931
1373.948
1401.979

Figure S6

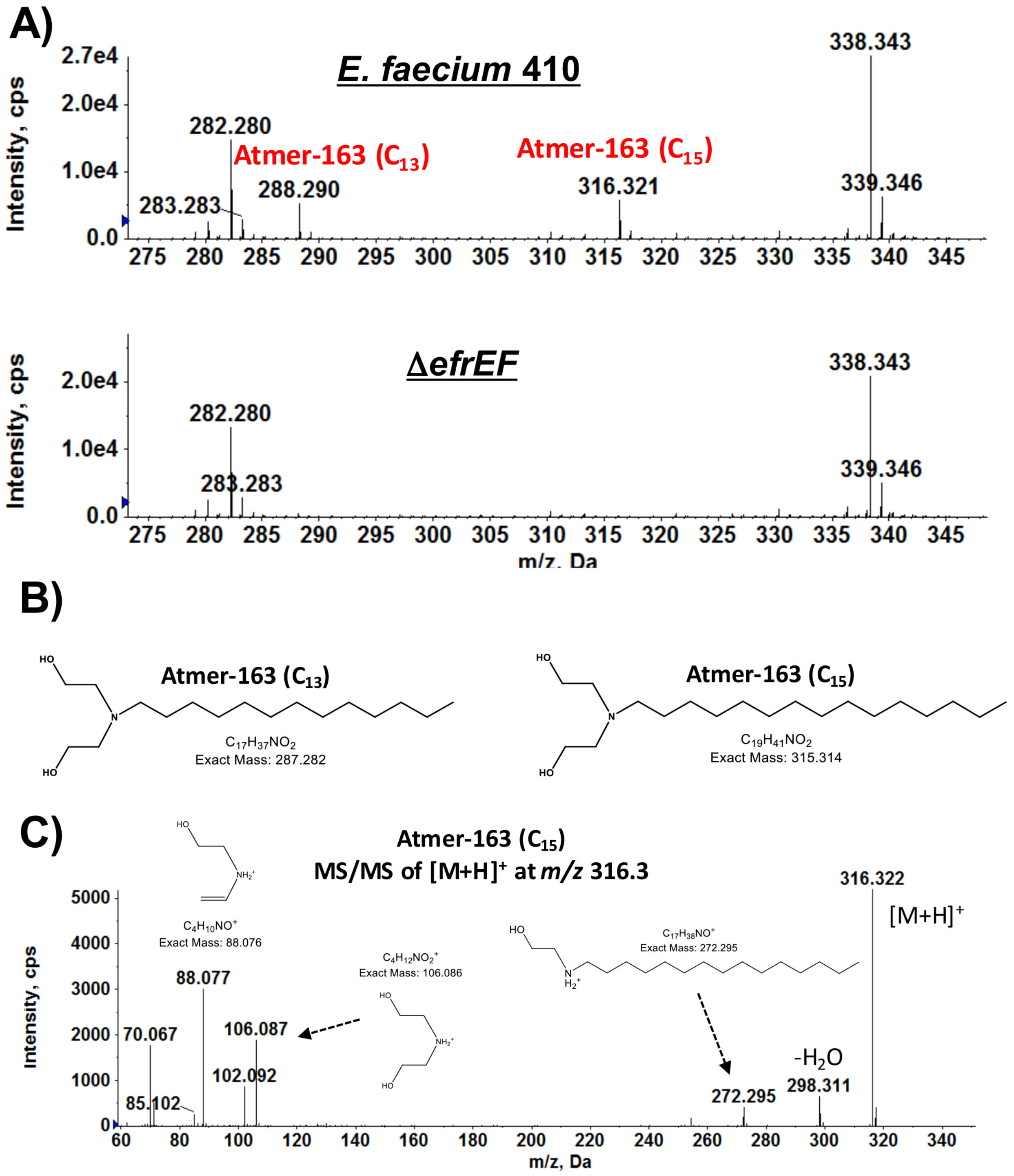

A)
E. faecium 410
Intensity, cps
2.7e4
2.0e4
1.0e4
0.0
338.343
282.280
Atmer-163 (C13)
Atmer-163 (C15)
288.290
316.321
339.346
283.283
275 280 285 290 295 300 305 310 315 320 325 330 335 340 345
ΔefrEF
Intensity, cps
2.0e4
1.0e4
0.0
338.343
282.280
339.346
283.283
275 280 285 290 295 300 305 310 315 320 325 330 335 340 345
m/z, Da
B)
Atmer-163 (C13)
HO
N
HO
C17H37NO2
Exact Mass: 287.282
Atmer-163 (C15)
HO
N
HO
C19H41NO2
Exact Mass: 315.314
C)
Atmer-163 (C15)
MS/MS of [M+H]+ at m/z 316.3
Intensity, cps
5000
4000
3000
2000
1000
0
HO
NH2+
C4H10NO+
Exact Mass: 88.076
C4H12NO2+
Exact Mass: 106.086
C17H38NO+
Exact Mass: 272.295
316.322
[M+H]+
88.077
70.067
106.087
102.092
85.102
-H2O
298.311
272.295
60 80 100 120 140 160 180 200 220 240 260 280 300 320 340
m/z, Da

Table S1. List of primers used in the study.

**1.Real time primers**	
410 *clpX* for	GCAAAACGCTCGTTAGCTGT
410 *clpX* rev	CCAGACCCTGTAGGTCCGAT
410 *liaX* for	CCCAGCATTTTTCGCAACGA
410 *liaX* rev	TGCATGGCAAGTCAGGTGAA
733 *clpX* for	AAACGCTCGTTAGCTGTTGC
733 *clpX* rev	CCCTGTAGGTCCGATCAAGC
733 *liaX* for	GCAGCAAACGGAAACGTGAC
733 *liaX* rev	CTCGATCTGGCTCCGTTCAG
410 *cmk* for	GGCTCATCCTTCGGTGTGAA
410 *cmk* rev	GCACTGTTTCCCAATACCGC
410 *cls*1 for	CCTGGGGTGGGCTTTGTATT
410 *cls*1 rev	TATCGCCCATGTCAGGATCG
410 *cls2* for	CAAATCGTGACAAGCGGACC
410 *cls2* rev	AGAGCCAACTTCAGTGCTTCA
410 *chtS* for	TCGCTACGAGATCGTTGGTG
410 *chtS* rev	GTTTCCGTGATCCGGCGTA
410 *chtR* for	GCTATGAACATGGGGGCAGA
410 *chtR* rev	ACGTAATGCCATTGTGGCTC
**3. ABC transporter**	
ABC transporter flankingarm1_for	ATGCAT**GAATTC**CCTCTAAAAAACTAAAATTCT
ABC transporter flankingarm1_rev	ATGCAT**GCATGC**GATAGACATATTATTCCTCCT
ABC transporter flanking arm2_for	ATGCAT**GCATGC**ATAGCGTAACAAACATTATCT
ABC transporter flanking arm2_rev	ATGCAT**GGATCC**TGGACAACACATATGATATTT
ABC complementation for	ATGC**GGATCC**GCGGATCGTTGATTTTGGCA
ABC complementation rev	ATGC**GGATCC**CCTGCCTCTTCCGTTCAACT
**4. Confirmation of mutations**	
ABC transporter for	GCTTGGGTCACTCCAATTGC
ABC transporter rev	CCGTCGTAATGAGGCAAACG
RelA for	GTGACAAAAGAAGAAATTTTAACG
RelA rev	CTATCCATTCGTCCGGCGTACACT
Hydrolase for	TGGGATGGCTAGATCGGCTA
Hydrolase rev	CGCTGCTTCTGTAGGGACAA
cmk for	TGGCAACGGTACTTTTCCCA
cmk rev	GTGCAGGGATCACCACTGAT
PhoU for	ATGGGGATGATGGTGAGCAG
PhoU rev	CTGCCCTTTTACGCGAATCG
Glycosyl transferase for	CCAAGTATCCCTGCTCCGAA
Glycosyl transferase rev	AAGGTGCGTATTCAGGTGCA

Table S2. Doubling time and Rifampin mutation frequency for the DAP strains vs. *E. faecium* 410 wild-type.

Strains	Average doubling time ± SD (mins)[a]	*P* value[b]	Average Rifampin mutation frequency ± SD (X 10^{-7})[a]	*P* value
***E. faecium* 410 wt**	29.27 ± 1.63		1.68 ± 1.75	
**DAP-A1**	37.82 ± 5.26	*0.028	3.16 ± 3.53	0.13
**DAP-A2**	42.67 ± 2.78	*0.013	3.23 ± 4.66	0.18
**DAP-B1**	38.48 ± 2.09	*0.014	0.615 ± 0.19	0.14
**DAP-B2**	29.12 ± 1.92	0.244	0.975 ± 0.52	0.25

[a]Average values and standard deviation (SD) were calculated from minimum n=3 independent trials.
[b]Significance value was calculated using one-tailed Student's *t* test. *, $P < 0.05$. *E. faecium* 410, *E. faecium* 1,231,410.
